# Acupuncture for pain and osteoarthritis of the knee: a pilot study for an open parallel-arm randomised controlled trial

**DOI:** 10.1186/1471-2474-10-130

**Published:** 2009-10-24

**Authors:** Harriet Lansdown, Katie Howard, Stephen Brealey, Hugh MacPherson

**Affiliations:** 1 Department of Health Sciences, University of York, York, UK; 2 Foundation for Research into Traditional Chinese Medicine, York, UK

## Abstract

**Background:**

There is some evidence that acupuncture for pain and osteoarthritis (OA) of the knee is more than a placebo, and short term clinical benefits have been observed when acupuncture is compared to usual care. However there is insufficient evidence on whether clinical benefits of acupuncture are sustained over the longer term. In this study our key objectives are to inform the design parameters for a fully powered pragmatic randomised controlled trial. These objectives include establishing potential recruitment rates, appropriate validated outcome measures, attendance levels for acupuncture treatment, loss to follow up and the sample size for a full scale trial.

**Methods:**

Potential participants aged over 50 with pain and osteoarthritis of the knee were identified from a GP database. Eligible patients were randomised to either 'acupuncture plus usual care' and 'usual care' alone, with allocation appropriately concealed. Acupuncture consisted of up to 10 sessions usually weekly. Outcome measures included Western Ontario and McMaster Universities (WOMAC) index with the sample size for a full scale trial determined from the variance.

**Results:**

From the GP database of 15,927 patients, 335 potential trial participants were identified and invited to participate. After screening responses, 78 (23%) were identified as eligible and 30 patients who responded most promptly were randomised to 'acupuncture plus usual care' (15 patients) and 'usual care' alone (15 patients). Attendance for acupuncture appointments was high at 90% of the maximum. Although the trial was not powered to detect significant changes in outcome, the WOMAC pain index showed a statistically significant reduction at 3 months in the acupuncture group compared to usual care. This was not sustained at 12 months. The sample size for a fully powered two-arm trial was estimated to be 350.

**Conclusion:**

This pilot study provided the evidence that a fully powered study to explore the longer term impact of acupuncture would be worthwhile, and relevant design features for such a trial were determined.

**Trial registration number:**

ISRCTN25134802.

## Background

Pain and osteoarthritis (OA) of the knee are accompanied with varying degrees of functional limitation and reduced quality of life[1] In adults aged 45 years and over, the most common site of peripheral joint pain is in the knee and the highest prevalence of knee pain is amongst women aged 75 and over[2] Established risk factors for knee OA include obesity, age, female gender, misalignment, and knee injury [3-5] Osteoarthritis of the knee is a serious and chronic condition. In an increasingly ageing and obese population, the prevalence and incidence of OA of the knee is predicted to rise[6]

Patients may present with symptoms of pain and stiffness, joint instability, crepitus, decreased function and mobility. Diagnosis is commonly made clinically in primary care, and may be confirmed by radiological tests. The mainstay of conventional treatment includes analgesia, physiotherapy and joint replacement if indicated. Whilst osteoarthritis has often been considered as progressive and incurable,[7] the disease has also been viewed as a metabolically dynamic, essentially reparative process that is potentially amenable to treatment[8]

Three recent high quality meta-analyses of pooled data from randomised controlled trials of acupuncture treatment for osteoarthritis of the knee provide consistent evidence that acupuncture is more effective than sham acupuncture when patients are blinded to the intervention they received [9-11]. For example in one review, Manheimer et al found effect sizes ranged between 0.35 (95% CI: 0.15 to 0.55) for short term outcomes and 0.13 (95% CI: 0.01 to 0.24) for long term outcomes[10] Only one of the three reviews pooled data comparing acupuncture to usual care alone in which patients were unblinded. For this comparison, statistically significant differences were again found but with larger effect sizes of 0.62 (95% CI: 0.49 to 0.75) for short term outcomes (up to 3 months) and 0.52 (95% CI: 0.39 to 0.66) for longer term outcomes (at 6 months)[10] It may be counter-intuitive that effect sizes were larger when acupuncture was compared to usual care (an active comparator), however it is consistent with findings across the field that sham acupuncture tends to be highly therapeutic[12] Despite this emerging data, the UK National Institute for Health and Clinical Excellence (NICE) guidelines published subsequently state that, "There is not enough consistent evidence of clinical or cost-effectiveness to allow a firm recommendation for the use of acupuncture for the treatment of osteoarthritis."[1]

There are a number of limitations to the existing evidence. Firstly, none of the studies answered the pragmatic question about the effectiveness of acupuncture as an adjunctive treatment to usual primary care as delivered in the UK. A second problem is that most trials tended to have short-term follow-ups, typically no more than 26 weeks, more commonly as little as a month. While significant longer-term effects of acupuncture when compared to usual care have been shown for headache over 12 months,[13] and for low back pain over 24 months,[14] there is inconclusive evidence of longer term benefits of acupuncture for osteoarthritis of the knee. Thirdly the evidence on cost-effectiveness is limited, with trial data from Germany,[15] but no trial data relevant to the UK context[1] The combination of these limitations makes it difficult to estimate health service benefits and costs that need to be considered as part of a NHS decision process.

Given the challenges of treating osteoarthritis of the knee, the NICE guidelines have clearly identified the need for further studies to evaluate non-pharmacological therapies with a focus evaluating longer-term and sustained clinical effects as well as cost-effectiveness[1] NICE guidelines have also identified the need concurrently to address quality of life issues and comorbid conditions that compound the effect of osteoarthritis[1] It is in this context that we have designed a pilot study to identify the key design features necessary for a full scale randomised control trial. Our key objectives were to establish potential recruitment rate, appropriate validated outcome measures, attendance levels for acupuncture treatment, loss to follow up and the sample size for a full scale trial.

## Methods

### Design

We conducted a pragmatic parallel two-armed randomized control trial (RCT) comparing 'acupuncture and usual care' to 'usual care' alone. This was a Phase II study based on the methodology set out in the Medical Research Council's guidelines for the evaluation of complex interventions[16] The pragmatic design builds on current evidence that there is a significant difference between acupuncture and sham acupuncture for this condition [9-11]. It addresses practical research questions raised by NICE regarding clinical and cost effectiveness in an NHS context[1] In practical terms this pragmatic trial design offers best value by providing clinical results that are immediately applicable to patients and providers. The cost- effectiveness data, based on a real-world comparison will aid policy and decision-makers.

### Participant Recruitment and Randomization

Participants were recruited from a York-based GP practice with a list size of 15,927 patients. A search of their database identified patients over 50 years old who had consulted their GP in the last 3 years with knee pain, using the READ codes of 'knee pain', 'knee joint pain', 'osteoarthritis of the knee', 'anterior knee pain', 'other knee injury', 'painful right knee' and 'arthralgia'. These classifications were used to capture the wider population of patients with clinical symptoms of OA of the knee, but no radiographically confirmed diagnosis[17] These patients were sent an information leaflet, consent form and a screening questionnaire. Patients were recruited if they reported experiencing ongoing pain and stiffness in their knee, but were not under cancer care review, currently receiving acupuncture, having had a knee or hip replacement, involved in any insurance claim or litigation related to their knee pain, or suffering from rheumatoid arthritis or haemophilia. Recruited patients were randomised by the York Trials Unit using a computer-generated (STATA) random allocation method. As participants were enrolled their pre-randomisation number was emailed to the Unit, and their post-randomisation number and the group allocation to emailed back by return. This ensured that the person recruiting participants was different from the person implementing the allocation sequence in accordance with CONSORT guidelines[18] The study gained ethical approval from the York Local Research Ethics Committee on the 6^th ^June 2006 (ref: 06/Q1108/30).

### Outcome measures

Our primary outcomes are to establish the patient recruitment rate, appropriate validated outcome measures, attendance levels for acupuncture treatment, loss to follow up and the sample size for a full-scale trial. Our clinical outcomes included the Western Ontario and McMaster's University Osteoarthritis Index (WOMAC)[19], Oxford Knee Score (OKS)[20], SF36 version 2[21] and EQ-5D[22] The WOMAC is used to measure dimensions of pain, stiffness and function in knee and hip osteoarthritis, and the higher the score the more severe impairment. The OKS is designed to measure function and pain and total score. A lower score indicates a better state of health. The SF36 and EQ5D were used to measure health status and quality of life with higher scores indicating a better level of functioning. All data were collected by post at baseline, and 1, 3 and 12 months post randomisation, with reminders sent out as necessary for the first two of these follow-ups.

### Intervention

Patients allocated to the acupuncture treatment group were referred to one of 5 acupuncturists who were members of the British Acupuncture Council (BAcC), with a minimum training of three years full-time and at least three years post qualification experience. Acupuncture practitioners were advised they could see these patients for up to 10 treatments as necessary. The participating acupuncturists agreed to follow an adapted protocol developed for the treatment of depression[23], with a focus on the use of clinical judgment drawing on a specified range of theoretical frameworks This flexible approach meant that the numbers of needles inserted, depth of needle insertion, needle responses elicited, needle stimulation used, needle retention time and needle type varied. Treatments were usually weekly and key treatment details were recorded in a treatment log, including theoretical frameworks, acu-points used, and any additional components of treatment provided. Adverse events reported by patients to practitioners were recorded after each treatment.

Both groups received 'usual care', which included any appointments, medications (prescribed or over the counter) and interventions sought by participants from any health practitioner. Data on all usual care treatments received by both groups were collected using follow-up postal questionnaires at 3 and 12 months.

### Statistical analysis

Using SPSS version 15, data were analysed on an intention to treat basis. A regression model, with baseline outcome measure as the covariate, was used to assess changes over time between the two treatment groups in pain, function and other health related outcomes. The standard deviation of the outcome variable was used to calculate the sample size needed for a full scale trial using PS: Power and Sample Size Calculation .

## Results

### Recruitment rate and baseline data

**Figure 1:**
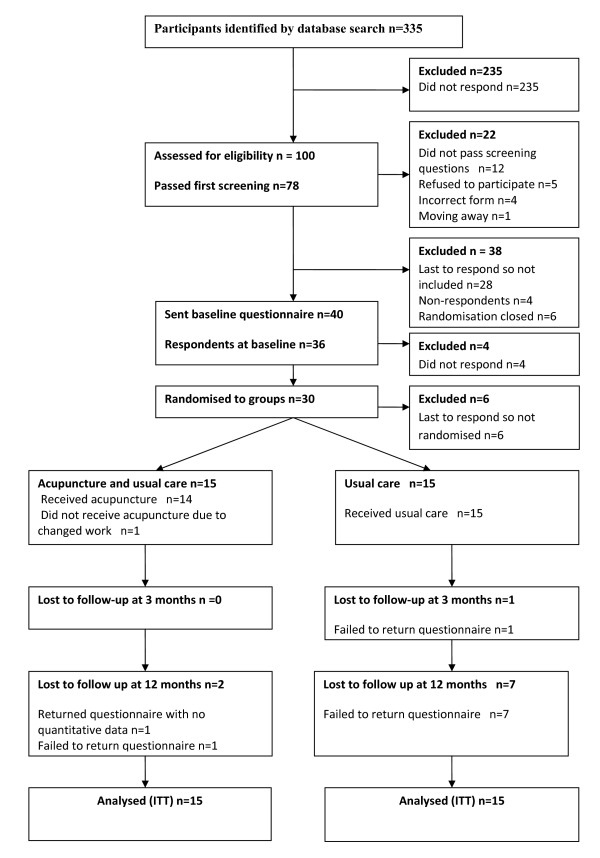
**Flow chart displaying details of eligible patients**.

From the database of one GP practice, 335 patients were identified as potentially eligible to enter the trial. On mailing these patients, 78 returned screening questionnaires and consent forms, and were found to be eligible (see Figure 1.) The 40 people who responded most promptly were sent the baseline questionnaire, on the basis that they were most likely to complete the trial and return questionnaires. Of the 40 patients who were sent the questionnaire, 36 people returned their forms. The first 30 patients to reply were selected and randomised into two groups: 'acupuncture and usual care' and 'usual care'. The baseline data collected prior to randomization are presented in Table 1.

**Table 1:** Baseline Characteristics of Patients

**Characteristics**		**Acupuncture**	**Control**	**Total**
**Mean Age**		62.9 (8.0)	64.2 (8.5)	63.5 (8.2)

**Female Sex (%)**		9 (60)	9 (60)	18 (60)

**Ethnicity (%)**	White	15 (100)	15(100)	30(100)

**Employment History (%)**				

	Employed part-time	0 (0)	1 (7)	1 (3.3)
	
	Employed full-time	6 (40)	2 (13)	8 (27)
	
	Self employed	1 (6.9)	0 (0)	1 (3.3)
	
	Retired	7 (47)	11 (73)	18 (60)
	
	Not employed but seeking work	1 (6.9)	0 (0)	1 (3.3)
	
	Not employed/not seeking work due to ill health	0 (0)	1 (7)	1 (3.3)

**Education**				

	Not in full time education (%)	15 (100)	15 (100)	30 (100)
	
	Age when leaving full time education (SD)	18.1 (7.4)	15.5 (1.3)	16.8 (5.4)
	
	Since leaving school/college/uni have had more part-time further/higher education (%)	7 (47%)	4 (27%)	11 (37%)

**Mean OKS (SD)**	30.93 (9.32)	30.6 (9.30)	30.77 (9.15)	

**Mean WOMAC Scores (SD)**				

	Pain 0-20	7.33 (2.82)	7.40 (3.66)	7.37 (3.21)
	
	Stiffness 0-8	3.13 (1.46)	3.8 (1.57)	3.47 (1.53)
	
	Function 0-68	20.53 (12.71)	26.27 (13.98)	23.4 (13.45)
	
	Global 0-96 0 = best health	31 (15.65)	37.47 (18.18)	34.23 (16.99)

**Mean SF36 Scores (SD)**				

	Physical Functioning	49.59 (26.57)	48.33 (24.54)	48.96 (25.14)
	
	Social Functioning	71.76 (25.21)	70 (23.53)	70.83 (23.97)
	
	Role Physical Functioning	62.5 (28.35)	52.92 (25.43)	57.71 (26.9)
	
	Role Mental Functioning	76.67 (26.01)	68.89 (35.56)	72.78 (30.87)
	
	Mental Health	76.33 (15.41)	69.33 (18.7)	72.83 (17.21)
	
	Vitality	55 (18.63)	46.25 (23.95)	50.63 (21.55)
	
	Pain	53.07 (18.71)	51.33 (24.31)	52.17 (21.42)
	
	General Health	67.27 (16.95)	55.05 (19.03)	61.16 (18.76)

**EQ5D (SD)**		0.61 (0.24)	0.67 (0.15)	0.64 (0.2)

### Attendance levels and intervention data

Of the fifteen participants randomised, fourteen attended 136 appointments (90% of the 150 maximum). Treatments were provided by four acupuncturists, usually on a weekly basis. Twelve patients attended all 10 available sessions. One participant discontinued treatment after 7 sessions as she felt her knee was very sensitive and did not feel there was any improvement. A second participant discontinued treatment after 9 sessions because she felt that the acupuncture treatment had been sufficiently successful. The acupuncturists used theoretical frameworks that included Channel Theory (14/14), Qi & Blood and Body Fluids (12/14), Zang-fu Syndromes (8/14) and Pathogenic Factors (7/14). The number of acu-points used in each treatment session ranged from 4 to 24, with a mean of 12. The most frequently used points were SP 6, 9, and 10; ST 36; LIV 3 and 8; LI 4; GB 34 and 41; KID 6; SJ 5; and the extra point Xiyan. Stainless steel needles were used with a diameter varying from 0.2 mm to 0.28 mm, length from 25 mm to 50 mm, and depth of insertion from 3 mm to 30 mm. De qi was usually elicited, and a variety of stimulation methods were used, including tonification and reduction. Retention time for the needles varied from 10 to 30 minutes. Auxiliary treatments of moxibustion (3/14) and acupressure massage (3/14) were provided. Lifestyle advice was offered to 11 out of 14 patients and most commonly in relation to relaxation (8/14), diet (6/14) and exercise (6/14 times).

Data on usual care are presented in Table 2, which shows that GP and physiotherapy consultations were the most common type of additional healthcare, and medication and walking aid use remained at an equivalent level between groups and over time.

**Table 2:** Data on usual care at 3 and 12 months

**Self reported health care received over preceding last 12 months**					
**Location of consultations with health practitioners**					

		**Acupuncture**		**Control**	
		
		**Reported at 3 months n = 14**	**Reported at 12 months n = 13**	**Reported at 3 months n = 14**	**Reported at 12 months n = 8**

GP surgery					

	GP	3	9	6	3
	
	Nurse	0	1	0	0
	
	Physiotherapist	2	0	3	0
	
	Other	4	0	0	2

NHS hospital					

	GP	2	0	0	2
	
	Nurse	0	0	0	0
	
	Physiotherapist	3	0	5	0
	
	Other	0	0	0	0

Private health care professionals					

	GP	0	4	0	0
	
	Nurse	0	0	0	0
	
	Physiotherapist	2	0	0	3

Self reported use of walking aids for knee and medication use over the last 12 months					

Number who have used a walking aid for knee		5	5	4	3

Number who have used medication for knee		7	7	8	4

### Outcomes measured at 3 and 12 months

**Figure 2:**
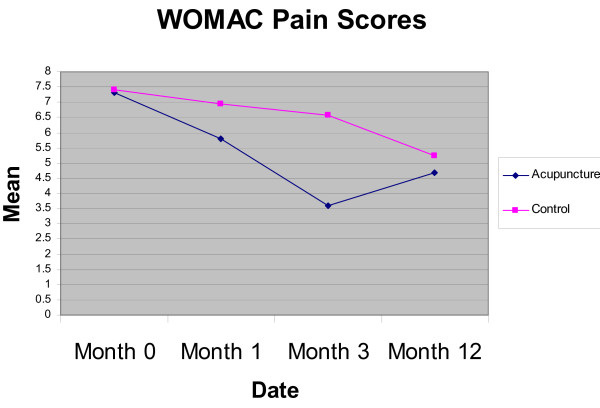
**Graph displaying Mean WOMAC Pain Scores (range 0 to 20)**.

**Figure 3:**
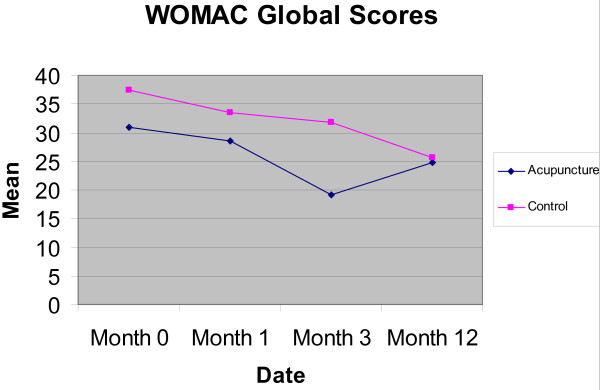
**Graph displaying Mean WOMAC Global Scores (range 0 to 96)**.

The outcomes for the WOMAC, OKS, SF-36 and EQ-5D at 3 and 12 months, with mean scores and SD, are shown in Table 3. The results (including 1 month data) are displayed in Figures 2 and 3 for the mean WOMAC pain and global scores. Adjusted differences in outcome using a regression model are shown in Table 4. Although the trial was not powered to detect significant changes in outcome, the WOMAC pain index showed a significant reduction [-2.62 (95% CI: -0.77 to -4.47)] at 3 months in the acupuncture group compared to usual care. This was not sustained at 12 months. No significant differences were observed on the OKS scale or the domains of the SF-36.

**Table 3:** Outcomes at three and twelve months

		**Acupuncture**	**Control**
**OUTCOMES AT THREE MONTHS**			

**Mean OKS (SD)**		23.07 (6.54)	26.07 (10.91)

**Mean WOMAC Scores (SD)**			

	Pain	3.6 (2.92)	6.57 (4.54)
	
	Stiffness	2.2 (1.78)	3.29 (1.86)
	
	Function	13.4 (12.12)	21.86 (11.99)
	
	Global	19.2 (16.52)	31.71 (17.5)

**Mean SF36 Scores (SD)**			

	Physical Functioning	60 (27.97)	52.14 (20.16)
	
	Social Functioning	78.33 (25.65)	71.43 (24.72)
	
	Role Physical Functioning	63.33 (28.63)	53.57 (29.08)
	
	Role Mental Functioning	76.11 (26.7)	76.79 (24.06)
	
	Mental Health	75 (14.64)	70 (21.99)
	
	Vitality	61.67 (18.13)	49.52 (17.01)
	
	Pain	68.93 (24.01)	59.57 (24.63)
	
	General Health	71.33 (18.62)	58.62 (21.27)

**EQ5D (mean)**		0.71 (0.26)	0.66 (0.25)

**OUTCOMES AT TWELVE MONTHS**			

**Mean OKS (SD)**		24.5 (7.5)	28.1 (9)

**Mean WOMAC Scores (SD)**			

	Pain	4.7 (2.3)	5.3 (3.9)
	
	Stiffness	2.7 (1.6)	2.8 (1.6)
	
	Function	17.4 (13.9)	17.6 (12.6)
	
	Global	24.8 (17.1)	25.6 (17.6)

**Mean SF36 Scores (SD)**			

	Physical Functioning	54.2 (29.5)	55.6 (24.9)
	
	Social Functioning	81.3 (20.3)	76.6 (20.5)
	
	Role Physical Functioning	71.4 (25.21)	57.8 (27.9)
	
	Role Mental Functioning	79.2 (26.95)	67.7 (18.6)
	
	Mental Health	73.1 (17.02)	65.0 (19.1)
	
	Vitality	58.2 (13.11)	46.9 (17.4)
	
	Pain	65.2 (22.3)	65.9 (17.3)
	
	General Health	67.7 (18.7)	62.4 (4.2)

**EQ5D (mean)**		0.66 (0.24)	0.63 (0.19)

**Table 4:** Adjusted differences in WOMAC scores comparing acupuncture to usual care at 3 and 12 months, using a regression model with baseline WOMAC score as a covariate

**WOMAC Index**	**Month**	**Adjusted means**		**Adjusted difference**	**95% CI**	**P value**
		**Acupuncture**	**Control**			

Pain	3	3.77	6.39	-2.62	-0.77 to -4.47	0.007
	
	12	4.38	5.76	-1.38	0.8 to -3.56	0.200

Stiffness	3	2.49	2.98	-0.49	0.56 to -1.54	0.346
	
	12	2.69	2.75	-0.58	1.3 to -1.4	0.930

Function	3	15.55	19.49	-3.94	3.4 to -11.3	0.281
	
	12	16.81	18.18	-1.36	8.7 to -11.4	0.778

Global	3	22.27	28.43	-6.17	3.1 to -15.4	0.184
	
	12	23.83	26.78	-2.94	9.5 to -15.4	0.624

### Loss to follow up

The loss to follow up in the 'acupuncture plus usual care' group was 0% at 3 months (n = 0) and 13.3% at 12 months (n = 2). It was higher in the 'usual care' group, at 6.7% at 3 months (n = 1) and 46.7% at 12 months (n = 7). We failed to send out reminders at the follow-up at twelve months.

### Adverse Events

No major adverse events were reported. Seven minor adverse events were reported (Table 5). The seven events out of a total of 136 treatments equate to adverse events occurring in 5.2% of treatments. No patients discontinued treatment as a consequence of adverse events.

**Table 5:** Minor adverse events reported by patients in the acupuncture group

**Type of acupuncture - related minor adverse event reported**	**Number of events**	**Did the patient discontinue treatment?**
Feeling faint	1	No

Unacceptable bruising	1	No

Worsening of existing symptoms with subsequent improvement	1	No

Worsening of existing symptoms with no subsequent improvement	1	No

Forgotten needle	1	No

Migraine	1	No

Pain at needle site	1	No

### Trial sample size and primary care list size for a fully powered trial

The WOMAC measure was chosen as the primary outcome for the sample size calculation for the full scale trial because it showed more sensitivity to change in this patient group than the OKS. We know from a previous study that an effect size of 0.39 on the WOMAC pain scale or 0.37 on the WOMAC function scale is needed to detect a minimally important change[24] We have selected the WOMAC pain sub-scale as our primary measure because of potential discordance in pain and function and because the overlap on the pain and function sub-scales may play a causal role in limiting the ability of the WOMAC function subscale to detect change[25] The mean square of the residual variance of the WOMAC pain score was found from our regression model to be 5.8, which can be converted to a standard deviation of 2.4. Given a minimum clinically important effect size of 0.39, the sample size required to detect this difference at 5% significance and 90% power is 139 patients in each arm of a two-arm trial. To allow for loss to follow up of 20%, a full-scale trial will require 350 patients in total.

Our evidence suggests that we should introduce a minimum WOMAC score of > or = 4 and at least 2 activities with at least moderate pain[26]. This will impact on our predicted recruitment rates, as only 63% (i.e. 49 patients out of the 78) of patients in this pilot met this cut-off score. Our GP practice had a registered list size of 15,900, therefore we estimate that to recruit 350 patients we will need a total primary care list size of approximately 115,000 patients.

## Discussion

### Key Findings

This pilot study has met key objectives by helping us identify useful information to assist the design of a full scale acupuncture trial for osteoarthritis of the knee. With regard to recruitment rate, we found that 23% of patients identified on a York-based GP database as eligible agreed to be recruited to the trial. With regard to outcome measures, we found the WOMAC scale to be more sensitive to change in this patient group than the OKS scale. Attendance at acupuncture sessions was high, with patients taking up on average 9 out of the 10 sessions available. We had a very poor (46%) loss to follow-up at 12 months, which is an area that will need better managing in a fully powered trial. We calculated that a sample size of 350 would be required to detect clinically relevant differences in this population.

Although the trial was not powered to detect significant changes in outcome, the WOMAC pain index showed a significant difference in favour of the acupuncture group at 3 months but this was not sustained at 12 months. Though no significant differences were observed in WOMAC function, stiffness or global scores, nor in the OKS, the trend was towards favouring acupuncture. The size of any difference may have been more accurately assessed if we had introduced a minimum baseline score for knee pain when screening for eligibility. A single positive result within this pilot must be interpreted with caution, given the number of statistical tests conducted, though it does reinforce the potential value of conducting a full-scale study.

### Comparisons with other studies

Our evidence is consistent with other studies that compared acupuncture to usual care for OA of the knee in which short term benefits at the end of treatment appeared to 'wear off' over time, including for example data from a meta-analysis[10] and from a large scale trial with a similar design from Germany[27] This pattern for OA of the knee is not consistent with recent primary care trials in the UK of acupuncture for headache over 12 months [13] and for low back pain over 24 months [14], which both showed increasing statistical and clinical differences between groups over the longer term. The reason for this may be that osteoarthritis of the knee is a progressive disease, whereas headache and back pain could be more likely to fully remit between episodes. However there remains a need for more primary data on the trajectory of changes in outcome over the longer term, a point emphasised by the recent NICE guidance and its associated recommendations[1]

### Limitations and strengths of study

The attrition rate was higher at 12 months than would be acceptable in a full scale trial, the reason being that no follow-up requests were made to those who did not respond to the initial request. For this pilot therefore, we have less confidence in the results at 12 months. For a full scale trial, it is recommended that a more systematic approach to collecting patient outcome data be put in place, including follow-up reminders and possibly financial incentives in the form of a voucher or payment to patients. We also note that by selecting the first patients to respond to our invitations, we recruited the more eager volunteers. This group may have higher adherence and follow-up rates than may be present in the primary care population.

Patient preference was not taken into account, and because it is likely that patients entered the trial with a preference, those allocated to usual care may have been more negative about their experience than those allocated to acupuncture. This may have biased the outcome in favor of the acupuncture group. However as the group sizes were very small the interpretation of these data are limited.

The pragmatic trial design is by necessity an open one. As a result, this trial does not answer the question as to the extent that the overall outcome was due to the 'specific components' of the intervention, or to the 'non-specific components', such as expectations of acupuncture. For this question, an explanatory trial design would be appropriate. However the cost would be the reduced applicability of the results to the general population of patients within primary care. Secondary gains of a pragmatic trial design for a full scale trial is that it can address the area of cost-effectiveness and provide relevant data for decision-making, whether by patients, service providers or policy makers. This pilot was based in one GP practice and one acupuncture clinic and therefore the results should be interpreted with caution.

### Future Research

There is a clear need for a full scale randomised controlled trial to confirm and extend the applicability of the tentative findings from this pilot at three months. There have been calls to establish whether any putative benefits at three months are sustained over the longer term. Monitoring patients in a trial over twelve months would help answer this question, as well as provide more robust data on cost- effectiveness, which would help reduce the dearth of UK related data as reported by NICE[1] To this end we recommend that resource use data associated with usual care be collected every three months, to reduce vulnerability to under-reporting. It would be worth considering other sources of data beyond self-reports, such as GP records for medication data. As part of "usual care", it would also be useful to capture the extent that "core" treatments, as proposed by NICE, such as advice and education, weight loss and exercise, are taken up. Our tentative findings seem to show that the effects of acupuncture might not carry-over beyond the end of treatment. If so, then it might be worth considering a trial design that includes the option of patients who have benefited receiving further top-up sessions after three months. A further dimension to this area of research is the need to explore any impact of acupuncture on quality of life. A new scale to measure quality of life in this population has recently been developed[20], and we recommend its use alongside the WOMAC index in a full-scale trial. Another area we suggest is monitored in a trial, which was identified by the NICE guidance as highly relevant to this patient group, is the impact of co-morbidities, such as anxiety and depression, that may compound the experience of osteoarthritis[1] There is a need to explore more fully the acceptability of acupuncture in this over 50s age group for which in-depth interviewing is probably the method of choice. Qualitative methods would also be useful in exploring the trajectory of recovery for patients with this condition.

## Conclusion

This study has shown that it is feasible to recruit patients to a primary care trial to receive acupuncture for osteoarthritis of the knee, and that the tentative findings support conducting a full-scale trial. The pilot data have led to an estimate of the sample required for a full scale trial as well as the expected recruitment rates. Recommendations have been made to assist in the design, with the emphasis on the need for data on longer term follow up, cost-effectiveness, quality of life, as well as the collection of qualitative data on acceptability and the trajectory of recovery in this patient group.

## Competing interests

The authors declare that they have no competing interests.

## Authors' contributions

HL conceived and conducted the study, including inputting and analysing the data; KH re-analysed the data and wrote the first draft of the paper; SB advised on the design, prepared the patient documentation, prepared the database, interpreted the results and helped with drafting the paper; HM helped with fund-raising, provided input into the design, supervised HL and KH, interpreted the results and redrafted the paper.

## Pre-publication history

The pre-publication history for this paper can be accessed here:


